# RETRACTION: The Influence of Direct Anterior Approach and Postero‐Lateral Approach on Wound Complications after Total Hip Arthroplasty: A Meta‐Analysis

**DOI:** 10.1111/iwj.70346

**Published:** 2025-03-17

## Abstract

**RETRACTION:** WuN. , MaJ. , XiongX. , LuoX. , MaX. , YangX. , WangW. , WangY. , WangZ. , and MaF. , “The Influence of Direct Anterior Approach and Postero‐Lateral Approach on Wound Complications after Total Hip Arthroplasty: A Meta‐Analysis,” International Wound Journal 21, no. 1 (2024): e14395, 10.1111/iwj.14395.37699722 PMC10784622

The above article, published online on 12 September 2023, in Wiley Online Library (http://onlinelibrary.wiley.com/), has been retracted by agreement between the journal Editor in Chief, Professor Keith Harding; and John Wiley & Sons Ltd. Following an investigation by the publisher, all parties have concluded that this article was accepted solely on the basis of a compromised peer review process. The editors have therefore decided to retract the article. The authors did not respond to our notice regarding the retraction.

**RETRACTION:** N. Wu , J. Ma , X. Xiong , X. Luo , X. Ma , X. Yang , W. Wang , Y. Wang , Z. Wang , and F. Ma , “The Influence of Direct Anterior Approach and Postero‐Lateral Approach on Wound Complications after Total Hip Arthroplasty: A Meta‐Analysis,” International Wound Journal 21, no. 1 (2024): e14395, 10.1111/iwj.14395.37699722 PMC10784622

The above article, published online on 12 September 2023, in Wiley Online Library (http://onlinelibrary.wiley.com/), has been retracted by agreement between the journal Editor in Chief, Professor Keith Harding; and John Wiley & Sons Ltd. Following an investigation by the publisher, all parties have concluded that this article was accepted solely on the basis of a compromised peer review process. The editors have therefore decided to retract the article. The authors did not respond to our notice regarding the retraction.

